# Live lecture versus video podcast in undergraduate medical education: A randomised controlled trial

**DOI:** 10.1186/1472-6920-10-68

**Published:** 2010-10-08

**Authors:** Benjamin E Schreiber, Junaid Fukuta, Fabiana Gordon

**Affiliations:** 1 Rheumatology Department, Royal Free Hampstead NHS Trust, London, UK; 2 Care of the Elderly, Bristol Royal Infirmary, Bristol, UK; 3 Statistical Advisory Service, Imperial College, London, UK

## Abstract

**Background:**

Information technology is finding an increasing role in the training of medical students. We compared information recall and student experience and preference after live lectures and video podcasts in undergraduate medical education.

**Methods:**

We performed a crossover randomised controlled trial. 100 students were randomised to live lecture or video podcast for one clinical topic. Live lectures were given by the same instructor as the narrator of the video podcasts. The video podcasts comprised Powerpoint™ slides narrated using the same script as the lecture. They were then switched to the other group for a second clinical topic. Knowledge was assessed using multiple choice questions and qualitative information was collected using a questionnaire.

**Results:**

No significant difference was found on multiple choice questioning immediately after the session. The subjects enjoyed the convenience of the video podcast and the ability to stop, review and repeat it, but found it less engaging as a teaching method. They expressed a clear preference for the live lecture format.

**Conclusions:**

We suggest that video podcasts are not ready to replace traditional teaching methods, but may have an important role in reinforcing learning and aiding revision.

## Background

Information technology has pervaded youth culture and the world of commerce and over the last five years is beginning to have a major impact on higher education [1]. Medical educators are always looking to find new tools with which to teach and recently these tools have been largely found within information technology. The technologies can be grouped into four categories: audio, video, computer-based and mixed. Podcasts are the predominant audio format. The term 'podcast' was coined in 2004 to refer to audio files downloaded automatically using Real Simple Syndication software and played on Apple's iPod™. However, now both audio and video files are downloaded and played on personal computers or on portable audio or video players [2,3]. Their popularity has grown in recent years as MP3 players and iPods™ have become so widely used.

Podcasts are now being used within professions allied to medicine, notably dentistry [4] and nursing [5], and, increasingly in undergraduate medical education. Other uses of information technology include video recordings of lectures which can be watched in medical libraries or over the internet [6,7] and various forms of computer based learning, such as medical school websites and educational software [8,9].

One of the emerging technologies in higher education is a combination of an audio recording of a lecture with video images of an accompanying Microsoft™ Powerpoint™ slideshow. This combination has been referred to as a video podcast [2], computer based learning [10] or audio/visual rich media presentations [11].

Podcasts have several potential advantages. Lecturers can use podcasts to augment their teaching and to teach without restrictions in time or place. Students appreciate the convenience of learning on the go and repeated learning while universities can use podcasts to offer learning beyond the physical boundaries of the campus [12].

From a pedagogic point of view, there are several features of podcasts which may enhance learning. Mayer, in his multimedia learning theory, suggested that successful learning using multimedia depends on recognising three features of learning: dual channels, limited capacity and active processing. The first assumption is that learners process visual and auditory information separately and that learning is enhanced when both are stimulated. The second assumption is that the capacity for learning in working memory is limited, and therefore the ability to pause a podcast or to repeat learning aids learning. The third assumption is that students learn best when they are allowed to interact with the learning material using active processing [13]. This interaction may involve the learner making an effort to make sense of multimedia presentations by paying attention, organizing information, and combining new information with previous knowledge from their long-term memories [14].

An additional pedagogic advantage of podcasts may be that they facilitate learning through social networks which increases the sense of connectivity of the learners. Siemens argues that "nurturing and maintaining connections is needed to facilitate continual learning" [15].

There are several potential downsides to podcasts. These include reduced interaction between lecturer and student which may hamper learning, the inability of the student to ask questions and the inability of the lecturer to gauge understanding from non-verbal cues and indeed from questions. As a consequence, the student may be less engaged in the learning and motivation may suffer.

We sought to compare information recall after live lecture and video podcast using a randomised controlled crossover trial. We also collected qualitative data on student preferences and experiences.

## Methods

We chose a cross-over randomised controlled trial to compare video podcasts to live lectures. Students were split into two groups. The first group attended a live lecture on arthritis and then a video podcast on vasculitis, while the second group attended a live lecture on vasculitis and then a video podcast on arthritis. Both groups were then assessed with a questionnaire to assess qualitative and quantative outcomes [Additional File 1].

Video podcasts consisted of slides designed on Powerpoint™ (Microsoft, WA, USA) with an audio recording superimposed using Soundstudio (Freeverse, NY, USA). They addressed rheumatology topics which the students had not been exposed to as undergraduate medical students in their first year of clinical medicine. One video podcast was concerned with arthritis whilst the other was concerned with vasculitis and both were approximately 15 minutes in duration. The video podcasts were posted onto a freely accessible website, http://www.podmedics.com. The video podcasts were delivered in a computer suite where each student was assigned their own computer with personal earphones. The video podcast was watched using Windows Media Player™ and the learner could pause or rewind the video podcast as required.

Two didactic lectures were also prepared which were matched to the video podcasts in content and duration. The same instructor presented the lectures and narrated the video podcasts on arthritis and vasculitis. The instructor was a fifth year medical student who was experienced in both lecturing and podcasting, having set up and delivered previous lectures in the voluntary evening course. Identical Powerpoint™ slides were used in the live lecture and video podcast. The duration of each lecture was 15 minutes.

Students were provided with written notes on both topics at the start of the teaching session to support both the live lecture and the video podcast.

Medical students in the first year of clinical study at Imperial College Medical School, London were invited to a voluntary, free additional teaching session accompanied by refreshments on a mid-term weekday evening. The students had no previous teaching in clinical rheumatology.

**Figure 1:**
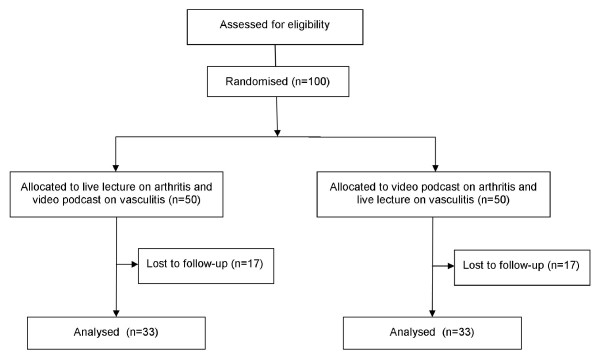
**Flow Diagram**. Flow diagram of student allocation.

The students were randomised as they entered into two groups each containing 50 students (Figure 1). The students in Group 1 were directed to the computer suite to complete the video podcast on arthritis whilst those in Group 2 attended a live lecture on the same topic.

Then the students in Group 2 were sent to the computer suite to complete the video podcast on vasculitis whilst the students in Group 1 attended a live lecture on the same topic. Again the same slides and lecturer were used in both. The groups were then brought together at the end to complete a questionnaire.

The assessment consisted of 19 multiple choice questions on arthritis and 15 multiple choice questions on vasculitis (see appendix). Students were asked whether they preferred lectures or video podcasts in terms of content, presentation, experience and comfort and ability to retain information. Information was gathered concerning the age, sex, ethnic origin and previous academic performance of each student.

The primary outcome was the score on the multiple choice questionnaire after each intervention. The study hypothesis was that knowledge after video podcasts would be not significantly different from knowledge after live lectures.

Sample size power calculation was performed for two sided 0.05 significance level to detect a difference of at least 22% with 80% power.

The statistical methodology used to analyse the data was Linear Mixed Models since the correlation between measurements on the same student needs to be taken into account. The modelling and estimation of the effects of interest was carried out by SPSS 16. The level of significance used was 5%.

The Charing Cross Research Ethics Committee advised that, in view of the nature of the project, it did not require approval by a research ethics committee.

## Results

### Baseline Data

100 students attended and were randomly assigned to groups 1 or 2. All students completed the assignment but only 66 completed the multiple choice questionnaire and feedback form.

The 66 returned questionnaires were equally distributed from groups 1 and 2 (33 each). The average age was 21.2 years (SD 1.36, range 20-26). There was no difference in age between the two groups (p = 0.72). 39.4% of the students were male in each group, with no difference between the groups.

In the self-reported free-text ethnicity, 16 in Group 1 and 13 in Group 2 gave their ethnicity as Caucasian, white or British. 13 in each group listed other ethnicities and 1 patient in each group left ethnicity blank.

The students were asked in which quartile they usually score on examinations. In all 30% rated themselves in the top quartile and 50% in the second quartile, 13% in the third quartile and only 7% in the lowest quartile. The mean self-reported academic score did not differ between the two groups (p = 0.51).

The students were asked to state their preferred method of learning. 92% of students preferred lectures and tutorials to computer based or self-directed learning.

### Students' rating of live lecture and video podcast

Students were asked to rate the content and presentation of the lecture and video podcast. The lecture content was rated as very good by 83% of students and rated as good by 17%. No students rated the lecture content as satisfactory, poor or very poor. Lecture presentation was rated as very good by 88%, as good by 11% and as satisfactory by 2%. None of the students rated it as poor or very poor.

The video podcast content was rated as very good by 62%, good by 30% and satisfactory by 8%. No students graded it as poor or very poor. The video podcast presentation was rated as very good by 55%, good by 39% and satisfactory by 6%. No students rated them as poor or very poor.

Comparison of the video podcast to the live lecture on presentation shows that the video podcast was rated lower than the lecture both on quality of presentation (mean 1.5 for video podcasts compared to mean 1.1 for live lecture on a 5 point scale, where 1 is the best and 5 is the worst, p < 0.001).

Comparison of the podcast to the lecture on content shows that the podcast content was rated a lower than the lecture presentation (mean 1.5 for video podcasts compared to mean 1.2 for live lecture on a 5 point scale, where 1 is the best and 5 is the worst, p = 0.002).

Thus, the students generally rated the podcast as having slightly inferior content and presentation compared to the live lecture.

In terms of learning and retaining new information, 54% found the lecture format much better, 40% found the lecture format a little better and only 6% found the podcast format a little better. No students found the podcast format much better.

In terms of comfort and pleasantness of the experience, 48% found the lecture format much better, 42% found the lecture format a little better and 11% found the podcast format a little better. No students found the podcast format much better.

### Knowledge Based Assessments

On multiple choice questions, the two groups performed similarly (Table 1).

**Table 1:** Results of Multiple Choice Questions

	Group 1	Group 2
Arthritis	*Podcast *88.8%	*Lecture *90.6%

Vasculitis	*Lecture *89.9%	*Podcast *86.7%

Comparison of the two groups shows that scores in arthritis questions were similar in the two groups (means 88.8% in group 1, 90.6% in group 2, p = 0.33 for comparison), as were scores on questions of vasculitis (mean 89.9% in group 1, 86.7% in group 2, p = 0.10 for the comparison).

Analysis using linear mixed models showed that there was no significant difference between the combined score on all questions after lectures (mean 90.2) and after podcasts (mean 87.8) (p = 0.15). Thus, the results from the analyses show that there is no statistically significant difference between the two methods for either of the two topics. Results did not differ on subgroup analysis by gender, historical exam results or preference for learning information.

### Qualitative Data

Students were also asked for their free text comments about the pros and cons of lectures and podcasts. Of 66 students, 55 students (83.3%) gave a written comment in response to these questions.

20 students commented on the convenience of a podcast as an advantage, writing that podcasts "don't involve travel", are "better if you want to stay in bed", "good if you can't attend the lecture" and are "fantastic for on-the-go learning".

17 students commented on the ability to control the playing of podcasts, which facilitates pausing to "write things down", "look things up on the internet", replay sections and to repeat the whole podcast. Students commented that with video podcasts one can "understand content before proceeding to the next section". One student wrote: "Being a slow learner, I find it useful to be able to rewind".

13 students however said that podcasts are less engaging. They felt that podcasts "require discipline". They "can be put off" and are "easy to put off". One is "less likely to do it" and there is "often no incentive". Students reported that once started on a podcast it is "hard to concentrate", "hard to concentrate and follow", "quite difficult to learn from" and "easy to clock off". The podcast is "not as engaging". It is "more dull" and so "sometimes you switch off". In addition podcasts, "whilst plausible, are susceptible to online interruptions". One is "less likely to finish" and "less motivated to study".

## Discussion

In this randomised crossover trial we found that students showed similar information recall after video podcasts and live lectures, but tended to prefer live lectures. Students appreciated the convenience and control over podcasts, but generally found them less engaging. They felt there was less motivation to learn with podcasts and that they were less likely to complete the teaching session.

Our study is, to our knowledge, the first cross-over randomised controlled trial to tackle this issue. This design allows us to establish that the students in the two groups are similar not only in their age, gender, stage of medical education but actually that they experience lectures in a similar way and that they achieve similar knowledge after a lecture. Other design strengths included testing the students only after the interventions, to avoid influencing the educational intervention with the test itself and keeping the two interventions as similar as possible using the same speaker, the same PowerPoint slides, and the same talk in the two different formats. The results showed that there was similar factual recall when comparing the traditional method of the didactic lecture against the online tutorial.

Our study adds to the existing literature on video podcasts. We explored a format which is becoming increasingly popular. Combining Powerpoint™ slides with an audio recording of the lecture can be done using standard software supplied with most computers and without use of a video camera. This format has been used in undergraduate and postgraduate courses in Marine Sciences in the UK, where it was used widely by students, particularly for revision and preparation for assessments [2]. Our qualitative findings mirror those of Parson in psychology undergraduate students. She found that students preferred podcasts accompanied by the lecturer's slides but that students felt that podcasts can only supplement traditional lectures, not replace them [16]. A similar conclusion was reached in other studies [2,17].

Randomised controlled trials have been used before to compare video podcasts to live lectures. One study addressed teaching of evidence based medicine in a medical school in Birmingham, UK. They found similar knowledge gain in students randomised to live lectures and those randomised to video podcast with Powerpoint slides™ and audio voiceover but student preferences were not directly assessed [10]. In a smaller trial from Michigan State University 12 medical students were randomised to live lecture and 17 to video podcast. The video podcast included not only Powerpoint™ slides and an audio recording of the lecture but also a smaller video window of the lecturer explaining the accompanying slides. They found no significant difference in exam results between the two small groups. Students randomised to video podcast appreciated not having to travel to live lectures but faced technical difficulties in watching the video podcasts [18].

The qualitative data collected in this study is consistent with some previous studies. In another study carried out in the same medical school, despite similar knowledge gains students were less keen to engage with computer based teaching methods [19]. In a study of short audio podcasts listened to while travelling, students reported that podcasts were engaging, efficient and effective compared to a textbook [20]. Thus, it would seem that podcasts are generally more engaging than a textbook but less engaging than a live lecture.

Strengths of our study include the crossover design which allows an internal control for differences between the two groups, the careful matching of the two interventions, excellent facilities for the lecture and video podcast and qualitative and quantative assessments.

Limitations of the study include possible selection bias as participation was out of hours and voluntary. The students self-reported their academic achievements, which may be unreliable. There was a significant drop out rate of students not filling in questionnaires with potential for response bias. Our interventions were brief and knowledge was assessed immediately after the intervention. A longer duration crossover trial with sequential knowledge assessments may be warranted.

## Conclusions

In our study most undergraduate medical students preferred a live lecture to video podcast, although knowledge gain was not significantly different. Students value video podcasts for convenience and ability to review, but find them less engaging than live lectures.

## Competing interests

The authors declare that they have no competing interests.

## Authors' contributions

BES participated in study design, statistical analysis and writing of the manuscript. JF participated in the study design, data collection and writing. FG participated in statistical analysis. All authors read and approved the final manuscript.

## Pre-publication history

The pre-publication history for this paper can be accessed here:

http://www.biomedcentral.com/1472-6920/10/68/prepub

## Supplementary Material

Additional file 1 **Questionnaire**. The questionnaire, including both knowledge assessment and qualitative questions. Click here for file
