# Supplementary material for: Live lecture versus video podcast in undergraduate medical education: A randomised controlled trial
Source: BMC Med Educ. 2010 Oct 8;10:68. doi: 10.1186/1472-6920-10-68 (PMC2958969; doi:10.1186/1472-6920-10-68)
Supplement: Additional file 1 — Questionnaire. The questionnaire, including both knowledge assessment and qualitative questions. [file 1472-6920-10-68-S1.DOC]

***Feedback form and Assessment Questions***

 
Thank you so much for completing this form. We are researching different teaching methods in medical education, and your full responses will be really helpful to us. Please use the free text at the bottom to give us your comments and feelings - they are very valuable.

 
**MULTIPLE CHOICE - SAY TRUE OR FALSE FOR EACH PART OF THE QUESTION**
 
1. The following disease is associated with ANCA antibodies

1. Wegener's Granulomatosis **TRUE / FALSE**
2. Rheumatoid Arthritis **TRUE / FALSE**
3. Ankylosing Spondylitis **TRUE / FALSE**
4. Churg Strauss **TRUE / FALSE**
5. Psoriatic Arthritis **TRUE / FALSE**

2. Which form of arthritis is associated with Heberden's nodes?

1. Psoriatic Arthritis **TRUE / FALSE**
2. Ankylosing Spondylitis **TRUE / FALSE**
3. Rheumatoid Arthritis **TRUE / FALSE**
4. Gout **TRUE / FALSE**
5. Osteoarthritis **TRUE / FALSE**

3. Which form of arthritis is usually symmetrical?

1. Psoriatic Arthritis **TRUE / FALSE**
2. Ankylosing Spondylitis **TRUE / FALSE**
3. Rheumatoid Arthritis **TRUE / FALSE**
4. Gout **TRUE / FALSE**
5. Osteoarthritis **TRUE / FALSE**

4. Which form of arthritis is associated with crystals?

1. Ankylosing Spondylitis **TRUE / FALSE**
2. Pseudogout **TRUE / FALSE**
3. Rheumatoid Arthritis **TRUE / FALSE**
4. Gout **TRUE / FALSE**
5. Osteoarthritis **TRUE / FALSE**

5. Which form of arthritis is associated with infection?

1. Rheumatoid Arthritis **TRUE / FALSE**
2. Reactive arthritis **TRUE / FALSE**
3. Septic Arthritis **TRUE / FALSE**
4. Gout **TRUE / FALSE**
5. Osteoarthritis **TRUE / FALSE**

6. Which disease is commonly associated with IgM antibody against IgG?

1. Psoriatic Arthritis **TRUE / FALSE**
2. Ankylosing Spondylitis **TRUE / FALSE**
3. Rheumatoid Arthritis **TRUE / FALSE**
4. Wegener's Granulomatosis **TRUE / FALSE**
5. Sjogren's Disease **TRUE / FALSE**

7. Which vasculitis is associated with large vessels?

1. Wegener's Granulomatosis **TRUE / FALSE**
2. Microscopic polyangiitis **TRUE / FALSE**
3. Giant cell arteritis **TRUE / FALSE**
4. Kawasaki disease **TRUE / FALSE**
5. Polyarteritis Nodosa **TRUE / FALSE**

**Please circle ONE of the following answers**:
 
How would you rate the lecture content:

1. Very good
2. Good
3. Satisfactory
4. Poor
5. Very poor

How would you rate the lecture presentation:

1. Very good
2. Good
3. Satisfactory
4. Poor
5. Very poor

How would you rate the podcast content:

1. Very good
2. Good
3. Satisfactory
4. Poor
5. Very poor

How would you rate the podcast presentation:

1. Very good
2. Good
3. Satisfactory
4. Poor
5. Very poor

In terms of learning and retaining new information:

1. I found the lecture format much better
2. I found the lecture format a little better
3. I found the podcast format a little better
4. I found the podcast format much better

In terms of comfort and pleasentness of the experience:

1. I found the lecture format much better
2. I found the lecture format a little better
3. I found the podcast format a little better
4. I found the podcast format much better

Please describe what you felt about your experience of the two formats - what were the pros and cons and when might you prefer one as opposed to the other:
_____________________________________________________________________________________________
 
_____________________________________________________________________________________________
 
_____________________________________________________________________________________________
 
Anything you particularly liked or disliked? Any other comments at all?
 
_____________________________________________________________________________________________
 
_____________________________________________________________________________________________ 
_____________________________________________________________________________________________

**Finally about you:**
 
Age ____
 
Gender ____

*We would like to see if the format preference is correlated with different cultural backgrounds, so thank you for answering the following question:*

Ethnicity ______________________________

*If you don’t mind answering the following question it would be very helpful. We would like to see if there is a correlation between this answer and format preference:*

Would you say you were academically, within your year group: **Please circle one**

1. My exam results are usually in the top quartile of the year (top ¼)
2. My exam results are usually in the second quartile of the year from the top (i.e. above average but not in top quarter)
3. My exam results are usually in the third quartile of the year (i.e. below average but not in the bottom quarter)
4. My exam results are usually in the bottom quartile of the year (bottom ¼)

If you had to choose - which of the following do you generally prefer? **Please circle one**

1. Lecture
2. Tutorial
3. Computer based learning
4. Self-directed learning – reading books/journals/notes
